# Principles of Calcite Dissolution in Human and Artificial Otoconia

**DOI:** 10.1371/journal.pone.0102516

**Published:** 2014-07-21

**Authors:** Leif Erik Walther, Alexander Blödow, Jana Buder, Rüdiger Kniep

**Affiliations:** 1 Department of Otorhinolaryngology & Head and Neck Surgery, University Medicine Mannheim, University of Heidelberg, Mannheim, Germany; 2 Department of Otorhinolaryngology, Helios Clinic Berlin-Buch, Berlin, Germany; 3 Max Planck Institute for Chemical Physics of Solids, Dresden, Germany; University of California, Merced, United States of America

## Abstract

Human otoconia provide mechanical stimuli to deflect hair cells of the vestibular sensory epithelium for purposes of detecting linear acceleration and head tilts. During lifetime, the volume and number of otoconia are gradually reduced. In a process of degeneration morphological changes occur. Structural changes in human otoconia are assumed to cause vertigo and balance disorders such as benign paroxysmal positional vertigo (BPPV). The aim of this study was to investigate the main principles of morphological changes in human otoconia in dissolution experiments by exposure to hydrochloric acid, EDTA, demineralized water and completely purified water respectively. For comparison reasons artificial (biomimetic) otoconia (calcite gelatin nanocomposits) and natural calcite were used. Morphological changes were detected in time steps by the use of environmental scanning electron microscopy (ESEM). Under in vitro conditions three main dissolution mechanisms were identified as causing characteristic morphological changes of the specimen under consideration: pH drops in the acidic range, complex formation with calcium ions and changes of ion concentrations in the vicinity of otoconia. Shifts in pH cause a more uniform reduction of otoconia size (isotropic dissolution) whereas complexation reactions and changes of the ionic concentrations within the surrounding medium bring about preferred attacks at specific areas (anisotropic dissolution) of human and artificial otoconia. Owing to successive reduction of material, all the dissolution mechanisms finally produce fragments and remnants of otoconia. It can be assumed that the organic component of otoconia is not significantly attacked under the given conditions. Artificial otoconia serve as a suitable model system mimicking chemical attacks on biogenic specimens. The underlying principles of calcite dissolution under in vitro conditions may play a role in otoconia degeneration processes such as BPPV.

## Introduction

Human otoconia represent biominerals, consisting mainly of calcite (>90 wt.-%) and a small amount of organic material (<5 wt.-%) [1]–[4]. Owing to their inertia they provide adequate sensory stimuli of the utricular and saccular maculae to detect linear acceleration and head tilts in relation to gravity [5], [6]. In humans, otoconia are the only calcite based biomineral which is involved in physiological processes.

The special materials feature of human otoconia is represented by an intergrowth of the calcite component with organic molecules, mainly glycoproteins and glycosaminoglycans, forming a composite system on the nanoscale, a so-called calcite based nanocomposite [3], [4].

Up to now there has been significant information on the role of the organic molecules in mammalian otoconia. Otoconin 90 is the principal soluble otoconial protein whereas otolin represents the principal insoluble matrix protein which is also present in fibrils interconnecting otoconia [7]–[12].

**Figure 1:**
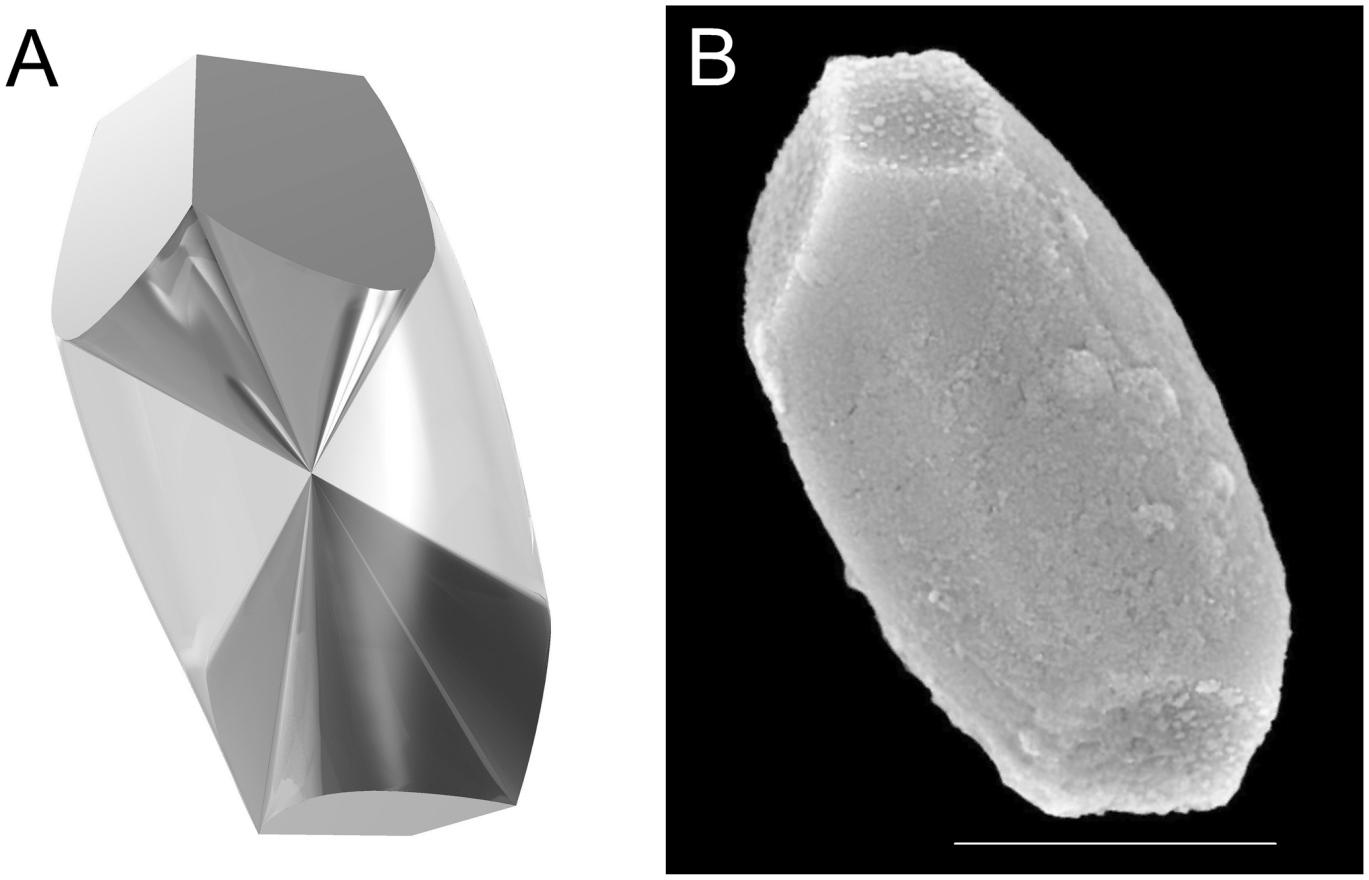
Inner structure *(A)* and outer shape *(B)* of a single human otoconium. *(A)* 3-D-model of the belly/branch-architecture [14], showing the belly (light-coloured) and the 3+3 branches (dark) meeting at the center of symmetry of the otoconium. The terminal planes representing the end faces of the branches at both sides of the otoconium are turned by 60° to each other. *(B)* ESEM-image of a single intact human otoconium. High vacuum (HV), 15 kV. Scale bar *(B)*: 5 µm.

Intact human saccular and utricular otoconia reveal a uniform architecture. Their outer shape is characterized by a cylindrical bulbous body with a slightly hexagonal equatorial contour and three terminal planes at both ends [1], [2], [13]. The mean size is around 10 µm [13]. The inner architecture of human otoconia consists of a dense structure forming 3+3 branches which are surrounded by a less dense, more porous belly-region demonstrating that there is no core/shell arrangement in mammalian otoconia, as was previously assumed [14], [15], [16]. The branches are more dense because of the highly ordered arrangement of nanocomposite units together with a parallel arrangement of fibrils. The belly appears to be less dense for reasons of a less ordered nanocomposite structure together with pores. Thus, the branch and belly regions differ in their volume densities. The inner belly/branch-architecture and the outer shape of human otoconia are presented in Figures 1A and B [14].

From the crystallographic point of view, human otoconia contain a 3-fold rotation axis in combination with a center of symmetry (so-called 3-fold inversion axis) and a mirror plane containing the 3-fold rotation axis, which causes tripling of the mirror plane. The branches including their terminal end faces are turned by 60 degrees, revealing an overall symmetry close to -3*m* (Figure 1 A and B) [3], [4], [14]. Human otoconia reveal Bragg-patterns (X-ray) of single human otoconia, which are representative for a single crystal of calcite [3], [4], [14].

Human saccular and utricular otoconia show gradual changes in morphology (degeneration) during lifetime [17]. Degeneration is associated with structural damage, a successive loss of otoconia material and related mass reduction. Fracture formation and detachment have been described in earlier post mortem investigations on otoconia from animals and humans, and also on human utricular otoconia from vital specimen [17], [18], [19], [20], [21]. It has also been shown that structural changes of human otoconia in the utricle showing degeneration such as fragment formation take place gradually during lifetime, which is assumed to be one factor of otoconia dislodgement into the endolymph causing benign paroxysmal positional vertigo (BPPV) [13]. BPPV is a common vestibular disorder leading to significant morbidity. Based on clinical evaluations BPPV has been reported to occur in >10% of the population in advanced age [22]. Furthermore, it has been proposed that otoconia dislodgement plays a role in peripheral vestibulopathies such as Ménière's disease (MD) and vestibular drop attacks [23], [24], [25]. However, information on the underlying etiological processes causing structural damage to otoconia has been limited up to now. Changes in the organic components were investigated in a recent study on animal otoconia (mouse) [26]. The authors showed a damage of the fibrils interconnecting the otoconia. It was assumed that the essential pathologic process leading to idiopathic (age-dependent) BPPV is probably due to demineralization of the subsurface layer, causing a loosening of the anchoring of interotoconial fibrils [26]. These effects are assumed to be responsible for detachment of otoconia in BPPV. In comparison, however, the inorganic component (calcite) is less investigated in relation to the underlying processes causing structural changes.

Since the discovery of biomimetic growth of artificial otoconia in gelatine gel matrices (calcite gelatine nanocomposits, CGC), which show the same chemical and structural characteristics as human otoconia, it has been possibly to detect morphological changes in great detail, as described in recent studies [3], [4], [14], [27]. From this it is concluded that artificial otoconia represent a suitable model system for the investigation of open biogenic problems under in vitro conditions [27].

The aim of this study was to investigate the main principles of morphological changes in human otoconia in dissolution experiments by exposure to hydrochloric acid, EDTA, demineralized water and completely purified water respectively. The chemical attacks by these agents were investigated with special consideration to the calcite component of otoconia. For comparison reasons artificial (biomimetic) otoconia (calcite gelatin composits) and natural calcite were used. Based on in vivo and in vitro findings, it is postulated that irreversible structural alterations (degeneration) are induced by chemical attacks such as pH changes, complexation reactions and changes in ion concentrations. Structural changes of pure inorganic calcite as well as human and artificial otoconia were investigated phenomenologically as a function of time by the use of environmental scanning electron microscopy (ESEM).

## Materials and methods

### Ethics

The study was conducted in conformity with the declaration of Helsinki 1975, revised in 1983, and was approved by the ethics committee of the University Medicine Mannheim, University of Heidelberg (2012-612N-MA). Written informed consent was obtained from all participants after the experimental procedure had been explained.

### Simulation of pH changes, complexation reactions and changes in ion concentrations

For dissolution reactions of artificial and human otoconia at low pH values (acidic range) hydrochloric acid (pH values 1.0–6.5) and for dissolution by complexation reactions the di-sodium salt of ethylenediaminetetraacetic acid (EDTA, c = 0.001–0.1 mol/L) were used, respectively. Changes in ion concentrations were simulated by the use of demineralized water (ion exchanger, c(Ca) = 5 µg/l; c(Na)<10 µg/l) and completely purified water (foreign ionic concentrations close to zero). The pH values were measured by the use of a MP220 system (Mettler Toledo, Germany) with InLab 410 Combination pH Electrode at the beginning and at the end of every experiment.

### Dissolution of pure calcite

In order to investigate details of the dissolution effects, rhombohedral faces of the bulk of a calcite single crystal (about 4.0 × 2.0 mm in size) were treated with hydrochloric acid, EDTA, demineralized water and completely purified water at room temperature. Surface changes were monitored in time steps and investigated by an environmental scanning electron microscope (ESEM) (FEI Quanta 200 FEGi, Eindhoven, Netherlands).

### Dissolution of artificial otoconia

Artificial otoconia (calcite gelatine nanocomposites, CGC) were grown by double diffusion into a gelatine gel (denatured collagen) matrix according to the methods described before [3], [4]. The gelatine gel was taken as the diffusion matrix, which at the same time was incorporated into the system forming the nanocomposite. Before starting the ESEM investigations the organic matrix surrounding the composite part of CGC was removed by washing with warm water in order to visualize structural changes in more detail. CGC were exposed to hydrochloric acid, EDTA, demineralized water and completely purified water for a defined period of time until dissolution was completed. Structural changes were studied by ESEM by the use of uncoated specimens in low vacuum (LV, 60 Pa). Acceleration voltages varied between 15–25 kV.

### Dissolution of human otoconia

Human otoconia were extracted from patients undergoing transmastoid labyrinthectomy for sporadic vestibular schwannoma as described recently [14]. Human utricles were identified and extracted with the maximum magnification of a surgery microscope (OPMI Vario/S 88 Carl Zeiss, Oberkochen, Germany) after removing the bony structures from the semicircular canals and the vestibule. Specimens were harvested after cutting out endolymphatic tissue with a beaver knife and were immediately fixed in ethanol (96%) for further structural investigations.

Samples lying in the gelatinous matrix were identified by light microscopy (Axioplan 2 imaging, Carl-Zeiss, Oberkochen, Germany) with 300-fold magnifications. After that, the samples were transferred to conductive (polycarbonate/graphite) foil discs (G3347, FEI/Philips) for investigations by ESEM. Groups of intact human otoconia as well as of otoconia in earlier stages of degeneration were identified at higher ESEM magnifications (up to >1:40,000). For investigations under high vacuum (HV) modes (2×10^−4^ Pa), some samples of human otoconia were coated with gold (Au) in order to obtain a reliable conductivity of the surface. Human otoconia were treated with hydrochloric acid, EDTA, demineralized water and completely purified water respectively. Monitoring of structural changes by ESEM was performed as described for artificial otoconia.

## Results

### Pure calcite

**Figure 2:**
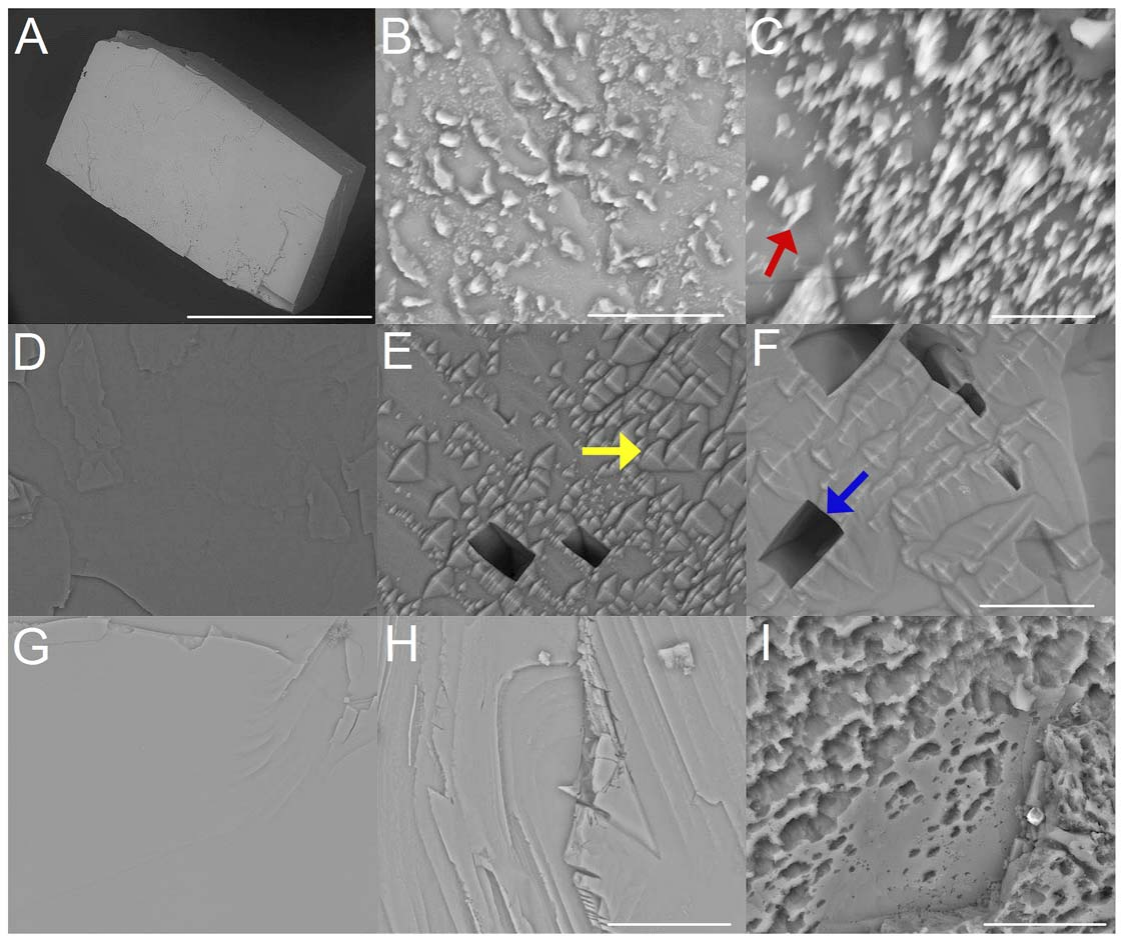
Rhombohedral faces of a calcite single crystals after exposure to hydrochloric acid (pH = 1) *(A,B,C),* EDTA (c = 0,107 mol/L) *(D,E,F)* and completely purified water *(G,H,I)*. *(A)* Calcite crystal before exposure. Time-dependent crystal surface changes after 120 min *(B)* and 180 min *(C)* treatment with hydrochloric acid. The red arrow in *(C)* shows dissolution induced spike like surface structures. *(D)* Crystal surface before EDTA exposure, after 60 min *(E)* and 180 min *(F)* EDTA-treatment. The yellow arrow in *(E)* marks pyramidal etch structures, the blue arrow in *(F)* shows a larger hole characterized by straight edges. *(G)* Calcite crystal before exposure, after 2.5 hours *(H)* and 27.5 hours *(I)* treatment with completely purified water. ESEM, low vacuum (LV), 15 kV. Scale bars (*A)*: 2 mm, (*B)*: 10 µm, *(C)*: 5 µm, *(F)* also for *(D,E)*: 20 µm, *(H)* also for *(G)*: 20 µm and *(I)*: 10 µm.

Treatment with hydrochloric acid leads to surface roughening and randomly distributed spike-like structures during the dissolution process (Figure 2 A,B,C). The rhombohedral faces of calcite show significant roughening effects with increasing exposure time leading to gradual reduction of material from the surface. Higher magnifications reveal the presence of randomly distributed spike-like structures on the surfaces in the course of the dissolution process. The strongest and most significant reaction is observed during treatment with EDTA, first causing randomly distributed depressions and roughening effects which finally develop into larger holes with straight edges (Figure 2 D,E,F). In addition, characteristic etch-figures are present between the deep holes (Figure 2 E,F). In the presence of demineralized water or completely purified water the dissolution reaction takes mainly place at terraces and steps on the rhombohedral faces after 2.5 (Figure 2 H) and 27.5 hours (Figure 2 I), leading to a successive reduction of material which finally leads to a porous surface structure (Figure 2 G,H,I).

### Artificial otoconia

**Figure 3:**
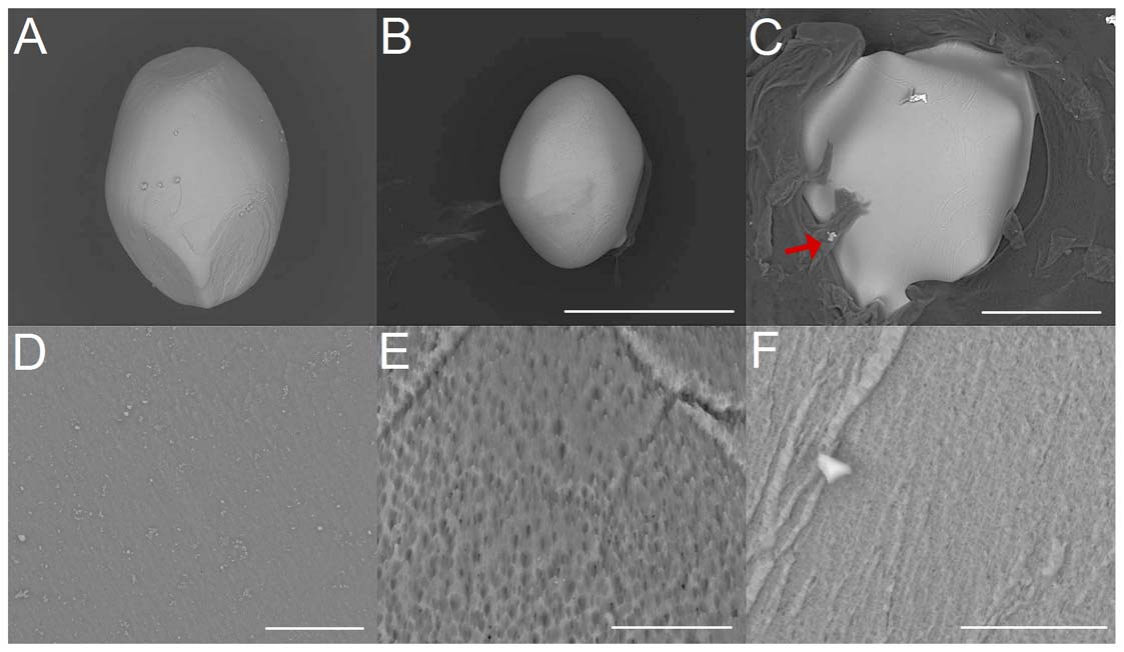
Dissolution of artificial otoconia by hydrochloric acid (pH 1) *(A,B,C)* and details of the dissolution effects (belly-area) *(D,E,F)*. *(A)* Intact artificial otoconium before treatment with hydrochloric acid. *(B)* Dissolution after 15 min and 70 min *(C)* exposure to hydrochloric acid. The particle in *(C)* is surrounded by significant amount of organic residue (see red arrow). *(D)* Surface before exposure. Dissolution after 15 min *(E)* and 70 min *(F)* exposure to hydrochloric acid. ESEM, low vacuum (LV), 15 kV. Scale bars in *(B)* also for *(A)*: 400 µm, *(C)*: 100 µm, *(D)*: 20 µm, *(E)*: 10 µm and *(F)*: 5 µm.

Exposure of artificial otoconia to hydrochloric acid (Figure 3 A,B,C) reveals a successive reduction in size under different pH values and exposure times, which appears to be almost constant in all directions of space (isotropic dissolution). Prior to the completion of the dissolution process, remnants are formed, characterized by roundedshapes (Figure 3 B,C). The organic component covering the specimen is left and does not seem to be actively involved in the dissolution process (Figure 3 C). With increasing pH value in the acidic range, the otoconia morphology changes during the dissolution process. The belly-area tends to be attacked first whereas the branches are dissolved at later stages. Close to the neutral-point, the dissolution process starts mainly in the belly-area and continues to extend to the branches at later stages. This kind of chemically induced changes in morphology, which is different in various directions of space is called anisotropic dissolution. Changes in the surface of the belly-area (Figure 3 D,E,F) show randomly distributed small pores and fissures of various sizes, which are smoothed out at later stages of dissolution.

**Figure 4:**
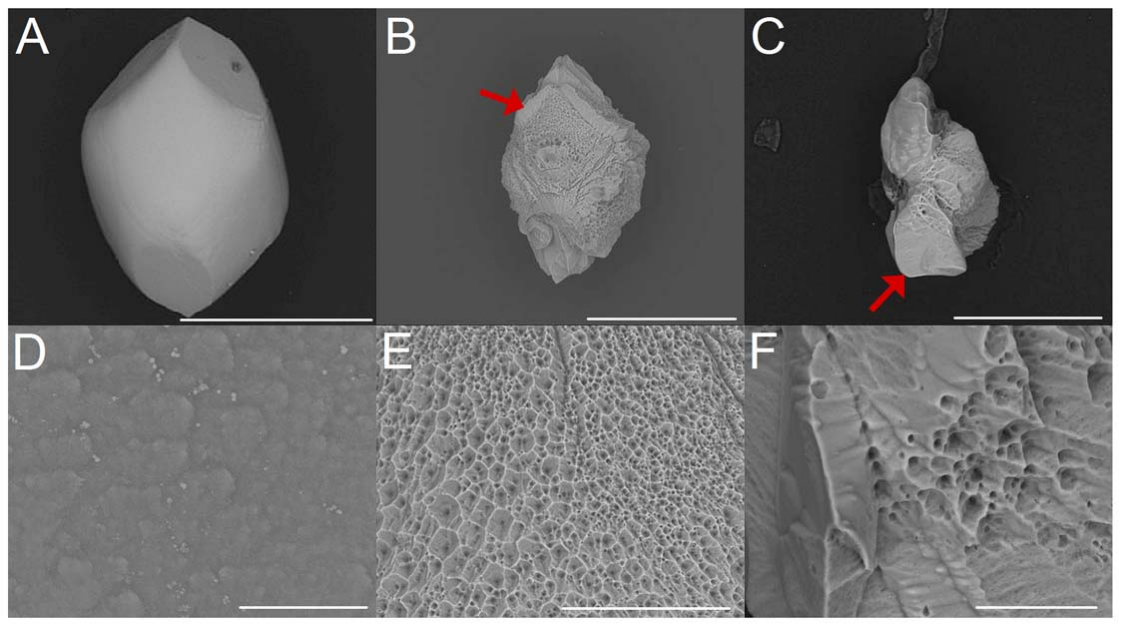
Dissolution of artificial otoconia after treatment with EDTA (c = 0,107 mol/L) *(A,B,C)* and details of the dissolution effects (belly-area) *(D,E,F)*. *(A)* Single otoconium before exposure to EDTA. Dissolution after 90 min *(B)* and 160 min *(F)* exposure. *(D)* Surface structure before EDTA treatment and after 30 min *(E)* and 140 min *(F)* exposure. The red arrows in *(B)* and *(C)* indicate one of the branches of the otoconium. ESEM, low vacuum (LV), 15 kV. Scale bars *(A)*: 400 µm, *(B)*: 200 µm, *(C)*: 100 µm, *(D)*: 10 µm, *(E)*: 50 µm, *(F)*: 20 µm.

In the case of EDTA exposure, artificial otoconia are immediately attacked in the belly-area by forming enlarged pores which develop into larger holes and fissures (Figure 4 A,B,C). This observation holds even for lowest EDTA concentrations (0.001 mol/L). After the center of symmetry of otoconia is reached, this area serves as the predilection site for fragment formation (Figure 4 C). These fragments, which contain parts of the more dense branches, are dissolved only at a later stage of reaction. Remnants consist mainly of parts of the branches. Dissolution after EDTA exposure shows a gradual reduction in size (anisotropic dissolution). Time-dependent surface alterations during exposure to EDTA reveal that the belly-surface is successively dissolved showing a gradual enlargement of pores and leading to fissures and larger holes (Figure 4 D,E,F).

**Figure 5:**
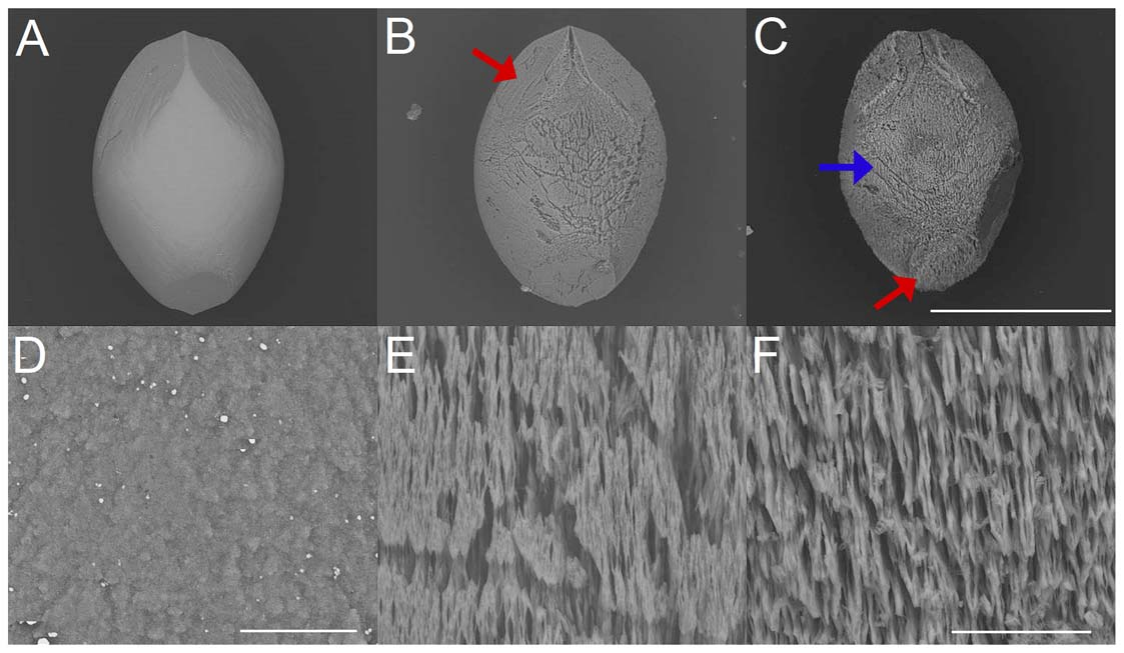
Dissolution of artificial otoconia and details of structural changes (belly-area), by treatment with demineralized water. *(A)* Single artificial otoconium before treatment. Dissolution after 110 hours *(B)* and 200 hours *(F)* exposure. The red arrows in *(B)* and *(C)* show the position of one of the branches. The blue arrow in *(C)* points to the belly-area. *(D)* Surface of the belly-area before exposure and after 110 hours *(E)* and 200 hours *(F)* treatment. ESEM, low vacuum (LV), 15 kV. Scale bars *(C)* also for *(A*–*B)*: 300 µm, *(D)*: 10 µm, *(F)* also for *(E)*: 20 µm.

The dissolution processes of artificial otoconia in demineralized water and completely purified water (Figure 5 A,B,C) reveal similar characteristics to EDTA treatment but proceed rather slowly and are more pronounced in the belly-area (moderate anisotropic dissolution). Surface changes (Figure 5 D,E,F) start with the formation of pores and fissures. Further dissolution leads to substructures in more or less parallel orientation.

Owing to their bigger size (up to 500 µm in length) the dissolution of artificial otoconia takes up to several hours compared with periods of minutes for human specimen (mean size up to 25 µm in length).

### Human otoconia

**Figure 6:**
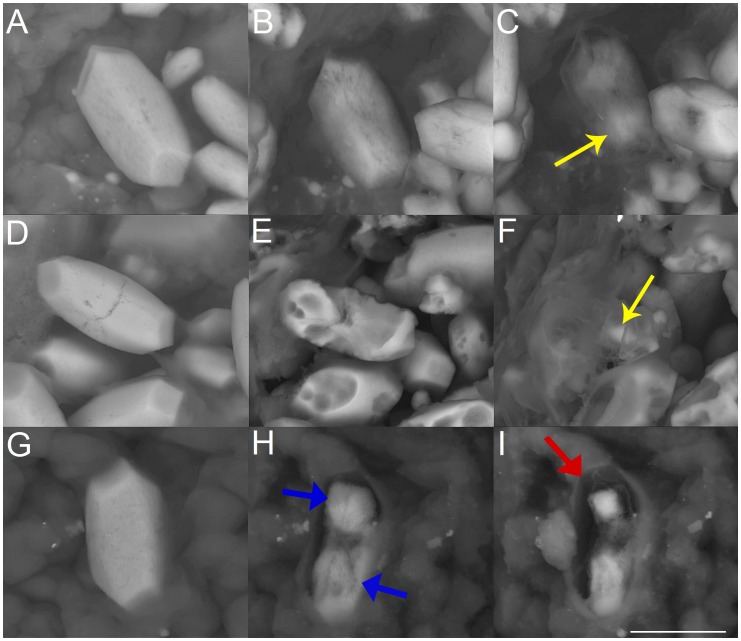
Dissolution sequences of human otoconia by treatment with hydrochloric acid (pH = 5) *(A,B,C)*, EDTA (c = 0.0025 mol/L) *(D,E,F)*, and completely purified water *(F,G,H)*. *(A)* Human otoconium before exposure to hydrochloric acid. Dissolution sequence 9 minutes *(B)* and 15 minutes *(C)* after exposure. The yellow arrow in *(C)* points to the more dense branches. *(D)* Human otoconia before treatment with EDTA and after 6 min *(E)* and 14 min *(F)* EDTA exposure. The yellow arrow in *(F)* shows that parts of the branches are left as remnants in later stages of dissolution. *(G)* Human otoconia before exposure to completely purified water. Dissolution 6 minutes *(H)* and 10 minutes *(I)* after exposure. The blue arrows in *(H)* indicate remnants of the branches. Red arrow in *(I)*: The organic component of otoconia keeps the original shape of the specimen. ESEM, low vacuum (LV), 15 kV. Scale bar *(I)* also for *(A*–*H)*: 5 µm.

In the case of hydrochloric acid the dissolution characteristic is shifted from isotropic in direction of anisotropic with increasing pH within the acidic range (Figure 6 A,B,C). The dissolution sequences reveal that the belly-are and the branch-regions are attacked in a similar manner. Parts of the branches are still present at later stages of dissolution.

EDTA exposure (Figure 6 D,E,F) leads to a chemical attack which starts in the belly-region and proceeds gradually. When the center of symmetry of otoconia is reached residues of the branches are still present. This dissolution leads to the formation of fragments of otoconia and remnants, which are part of the branches. The organic component of otoconia keeps the shape of the former composite (Figure 6 F).

In the case of demineralized water and completely purified water (Figure 6 G,H,I) a more anisotropic characteristic of dissolution can be seen. The structure of the organic component within the human otoconia specimen remains nearly unaffected and keeps the otoconial shape even after complete dissolution of the inorganic component (calcite) (Figure 6 H,I).

## Discussion

The results of this study show, that human and artificial otoconia reveal gradual dissolution phenomena caused by chemical attacks under in vitro conditions. Changes in the pH, ionic shifts and complexation reactions can be identified as the possible underlying principles causing structural changes. Since pure calcite is attacked chemically as well, it can be concluded that structural changes in human and artificial otoconia are caused by chemical interactions with the calcite component.

Human otoconia stay intact under in vivo conditions as long as the surrounding endolymph is saturated with regard to cations and anions which keep the equilibrium conditions for long-time stability of otoconia constant.

With regard to the inorganic main component of otoconia (calcite), the solubility product of CaCO_3_ defines the ion concentrations of Ca^2+^ and CO_3_^2−^ which are needed for stability reasons within the surrounding solution (endolymph) under the given conditions. In order to produce instability of otoconia, the Ca^2+^ and/or the CO_3_^2−^ ions or strictly speaking the solubility product, concentrations have to be changed within the surrounding medium. Upon such a change, the dissolution phenomena of otoconia are started. The CO_3_^2−^concentration can be reduced by protons (H^+^, H_3_O^+^) and the formation of HCO_3_^−^ which will finally react during further acidification to release CO_2_. The Ca^2+^ concentration is easily reduced by complexation reactions, preferably by the use of multidentate complexing agents such as EDTA. Finally, the exchange of the surrounding ion-containing solution for completely demineralized water will cause dissolution of the calcite component of otoconia until the equilibrium concentration of Ca^2+^ and CO_3_^2−^ is reached.

**Figure 7:**
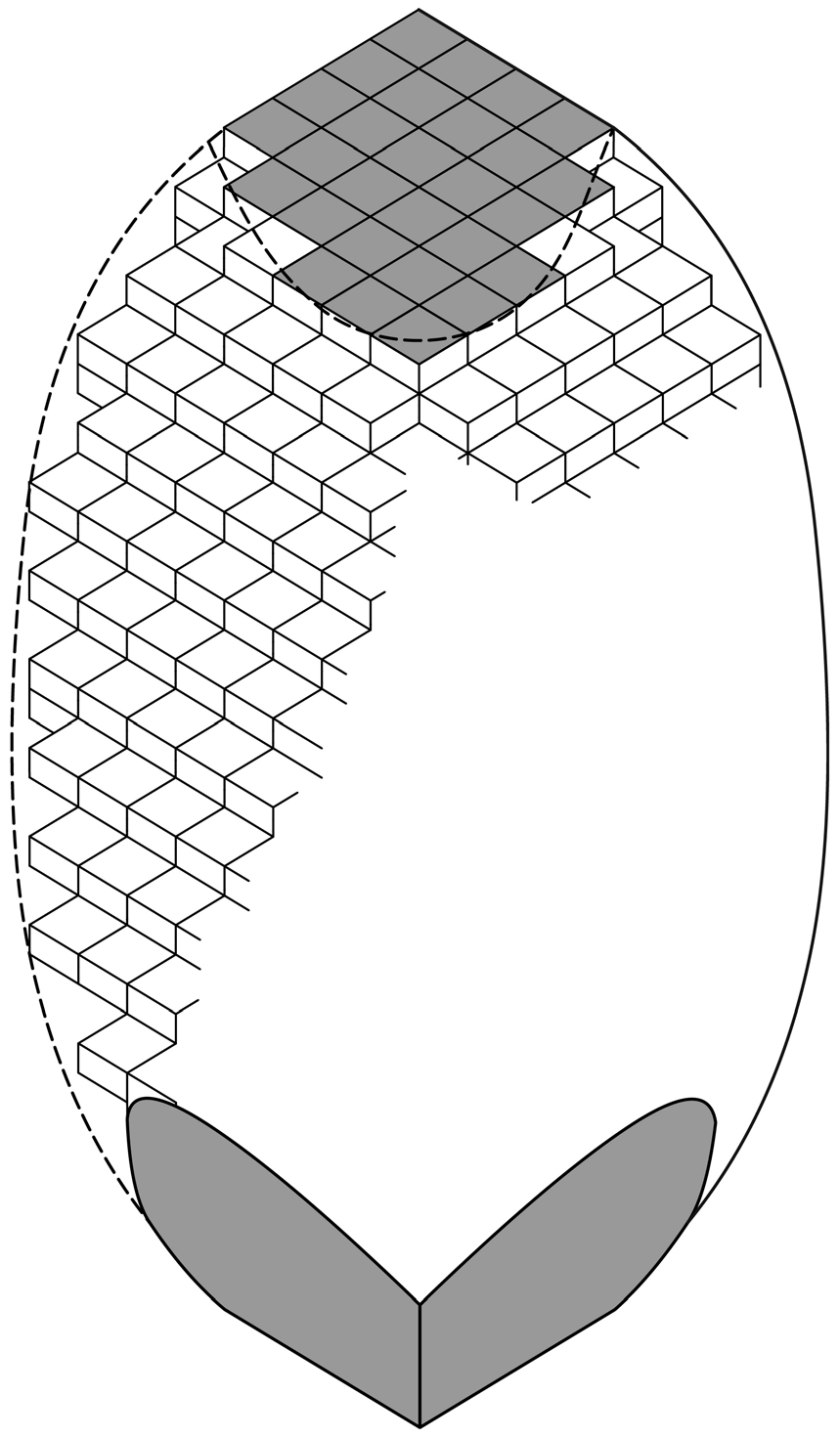
Schematic sketch of the calcite nanosubstructure of the surface of a single otoconium. The rhombohedral faces (dark) represent the end faces of the branches. The ellipsoidal shape of the belly-area, also built from rhombohedral subunits on the nanoscale, is dominated by structural steps indicating the increased surface roughness compared with the planar rhombohedral faces. The organic fibrils integrated into the composit are not shown. It is known, however, that fibrils in parallel orientation stick out perpendicular from the rhombohedral faces; within the belly-region a more irregular net of fibrils is stretched between the branches [3], [4], [14].

Chemical attacks on otoconia are mainly controlled by close contacts of the solvent molecules with the surface of the solid. The schematic sketch in Figure 7 shows, that the surface of otoconia is built of rhombohedral subunits in a three-dimensional periodic arrangement [3], [4]. The calcite particles within the nano-substructure of otoconia are not so perfectly arranged as shown schematically in Figure 7. The integration of the organic molecules in the composite system additionally causes mismatches between the calcite particles. This situation is called mosaic-arrangement and is characteristic for all kinds of biominerals acting as functional materials in living systems [4]. The dissolution reactions of otoconia are restricted to chemical interactions with the rhombohedral planes of calcite only. Two different features of the surface properties of otoconia are clearly seen i. the planar nature of the terminal end faces, and ii. the more rough and randomly patterned nature of the belly-surface, dominated by steps and related effects within the nano-substructure. Especially the steps on the belly surface give rise to close contacts with solvent molecules. Thus, the belly-area with its structural surface roughness is less resistant to chemical attacks, compared with the ordered and more plane terminal end faces. The real nature of otoconial material as an inorganic/organic nanocomposite is of crucial importance. The influence of pH shifts in the acidic range was already assumed by Johnson et al. [17] to cause structural changes in animal otoconia. Our dissolution experiments on human and artificial otoconia by acid attack (hydrochloric acid) under in vitro conditions (Figure 3 A,B,C and Figure 6 A,B,C) reveal that at low pH values the entire surface of otoconia is equally affected by volume reduction and the formation of rounded shapes even at the rhombohedral end faces. In final stages of dissolution rounded remnants occur which are assumed to consist of parts of the former branches. This kind of dissolution characteristic, in which otoconia do not reveal a preferred volume reduction in specific directions but show a more or less constant volume reduction in all directions of space is called isotropic dissolution. At higher pH values in the acidic range the belly-area, however, tends to be attacked first and a more anisotropic dissolution characteristic is developed.

The chemical interaction of calcite with EDTA represents a classical complexation reaction. EDTA is a six-dentate complexing agent and forms a very stable octahedral chelate arrangement with Ca^2+^ as the central ion. Because of the significant size of the organic molecule, the interaction of EDTA with the calcite component of otoconia starts mainly on the belly surface, leading to an anisotropic dissolution scenario. As can be seen from Figure 7, the belly surface is more roughened and less ordered compared with the planar terminal end faces and the more dense branches, which are only attacked in later stages of the reaction. The first observations made during this kind of anisotropic dissolution by EDTA are characterized by a roughening of the belly-surface, followed by an enlargement of pores and holes, which grow and penetrate into the inner structure of the belly [14]. After the center of symmetry of the otoconia is reached by progressing dissolution of the belly, fragments of the branches are formed without significant signs for rounding effects as observed in the case of acid attacks.

The same kind of pronounced anisotropy of the dissolution process of otoconia was only recently described by reaction with gentamicin under in vitro conditions [17]. In this context, gentamicin can be regarded as a five-dentate complexing agent for Ca^2+^, although a final proof for complex formation (structure of the complex) is still missing.

Earlier investigations in animals showed comparable structural changes of otoconia owing to systemic streptomycin administration [28], [29], [30], [31]. Aminoglycosides are able to enter the vestibular endolymph after systemic administration, leading to morphological changes of otoconia in the saccule and the utricle. Kusunoki et al. [32] demonstrated in temporal bones from patients who had aminoglycoside administration an increased prevalence of basophilic deposits of the cupula, which are assumed to contain degenerated otoconia particles (cupulolithiasis) causing BPPV [33], [34]. Furthermore, the assumption of a complexation induced BPPV following ototoxic otoconia damage is supported by observations made by Black et al. [35] who found an extremely high occurrence of BPPV (50%) in hospitalized subjects receiving ototoxic medications, mostly aminoglycosides. BPPV in all patients occurred independently of gender and age.

Finally we investigated morphological changes of otoconia in contact with demineralized water and completely purified water as the surrounding liquid (Figure 5 A,B,C,D,E,F and Figure 6 G,H,I). In a similar way to complexation reactions, the dissolution of calcite starts in the belly region leaving the branches as fragments. The anisotropic dissolution process is more pronounced in the case of the aqueous medium being completely free of foreign ions. When conventional demineralized water is used the reaction is slower. The dissolution reactions of otoconia with EDTA or water do not cause significant changes in pH values. This indicates that the structural decomposition of otoconia, in general, can run in a pH environment which is realized under living conditions.

The nano-structured inorganic component of otoconia (calcite) is extremely sensitive to chemical attacks which are caused by changes in ionic concentrations as well as the presence of foreign complexing agents in the surrounding aqueous medium (endolymph). Otoconia do not grow and/or regenerate during lifetime, a situation which is similar to mature teeth, which, however, are able to regenerate their surfaces by food ingestion. In addition, calcite is less stable to chemical attacks compared with apatite, the inorganic component of the teeth [36], [37].

Age-related alteration sequences in vital and post mortem mammalian otoconia have already been described and characterized by gradual reduction of material during lifetime [13], [19]. The origin of the morphological changes and their progressive development under in vivo conditions have remained unexplained in most cases up to now. Apart from ototoxic medications (aminoglycosides), changes in the electrolyte concentrations in the endolymph can also be induced by salicylates and some platinum-based anti-cancer drugs which might represent significant factors causing the various dissolution processes [27], [38], [39]. The alterations of otoconia morphology shown in our investigation represent a model study simulating extreme situations without consideration of realistic endolymphatic ion concentrations. Apart from the calcite component the organic minority component of the composite has also to be considered with regard to changes in structure, arrangement and positioning of otoconia. Recently, Andrade et al. [26] reported on the effect of structural changes of otolin in the fibrillar system of otoconia in animals (mice), which interconnects the otoconia and acts as an anchoring unit in the otoconial subsurface layers. These changes in the otolin structure are considered to cause otoconia detachment which is important in the etiology of BPPV. In fact, even minor changes in the otolin molecular structure cause significant differences in the interaction of the protein with calcium carbonate, as was shown by atomistic simulations only recently [40]. The organic fibril structure inside and outside of otoconia, however, did not seem to be affected in the course of our calcite dissolution experiments under in vitro conditions. There is only one aspect with regard to the organic fibril structure situated outside otoconia which may affect the dissolution experiments under certain circumstances. While the gelatinous layer which covers the surface of the larger artificial otoconia can easily be removed, as shown in the present study, this is sometimes not the case for the significantly smaller vital human specimens, which are more or less surrounded by an organic matrix. In the event of the former fibril net being irreversibly collapsed by intermediate low or high-vacuum treatment or other accidental (drying) events, this layer may act as a protective coating which slows down or even prevents further reactions. This scenario, however, has only been observed in rare cases. In general, the fibril nets around otoconia are permeable for aqueous solutions. Otoconia are composed of soluble and insoluble proteins. It has been shown that the insoluble organic component is extremely important in avoiding the dissolution of the calcite crystallites and provides the long-term protective mineralized feature [26].

The sum of the underlying attacks on otoconia and the resulting morphological changes during lifetime are designated as degeneration. As little is known even about the real function of intact otoconia, the same is true for degenerated specimen.

Investigations in several strains of mutant mice with a variable deficit or loss of otoconia in the saccule and the utricle, such as lethal milk mice, revealed a deficit using the linear acceleration vestibular evoked potential, which was correlated with the severity of otoconial loss. [41]. Abnormal ocular vestibular evoked myogenic potentials in subjects with BPPV indicate that abnormal function of the utriculus, is possibly due to damage to the utricular otolith membrane [42], [43], [44], [45].

In summary we have shown that irreversible structural alterations in human otoconia can be induced in vitro by chemical attacks such as pH changes, complexation reactions and changes in ion concentrations. The underlying principles of calcite dissolution under in vitro conditions might contribute to causing morphological changes such as assumed in BPPV.

## Conclusions

Our extended in vivo experiments have shown that the nano-structured calcite component of otoconia is extremely sensitive to chemical attacks which are caused by changes in ion concentrations as well as by the presence of foreign complexing agents such as gentamicin in the surrounding aqueous medium (endolymph). Reactions were performed by the use of hydrochloric acid, EDTA and demineralized water on artificial and human otoconia as well as on the rhombohedral faces of calcite single crystals. Because of their bigger size, artificial otoconia are perfectly suited for detailed investigation of degeneration effects as a function of time.
